# Lifelong Determinants of Cognitive Ageing: Insights from the British 1946 Birth Cohort

**DOI:** 10.1002/alz70856_097398

**Published:** 2025-12-24

**Authors:** Jonathan M Schott, Insight 46 study team

**Affiliations:** ^1^ Dementia Research Centre, UCL Queen Square Institute of Neurology, University College London, London, United Kingdom

## Abstract

**Introduction:**

An individual's risk of dementia results from a combination of their genetic background and life‐course exposures. Epidemiological studies suggests that ∼40% of dementia may be preventable worldwide, identifying 14 potentially modifiable risks operating at different stages across life. By contrast, genetic data suggests that 70%+ risk for AD is inherited. Birth cohorts where prospective life‐course exposure data can be combined with genetics and cognitive function and neurodegenerative biomarkers measured in later life provide a unique opportunity to explore factors that predispose to, and protect against, the development of brain pathologies and cognitive decline.

**Methods:**

The National Survey of Health and Development (NSHD) AKA the 1946 British Birth cohort is the world's longest continuously running birth cohort. Initially comprising *n* = 5,362 individuals born in mainland Britain in March 1946, the cohort has been prospectively studied in 27 data waves, including cognitive assessments at ages 8,11,15,26,43,53,60‐64 and 68‐69, and genetics/epigenetics at several time points. In 2015 (age 69) the Insight 46 neuroscience study was launched to investigate cognitive ageing: 502 individuals attended for an in‐person clinic visit including multi‐modal MR imaging, β‐amyloid PET, detailed neurological and cognitive testing, and fluid biomarker collection. *n* = 413 were seen for a second visit ∼ 2yrs later (+29 remotely), expanded to include cardiovascular testing and offer lumbar puncture. A third wave is underway (ages 77‐79): *n* = 350 of the original cohort are attending for a third visit including tau PET; *n* = 300 members of NSHD for a first visit including β‐amyloid PET. Enhancements include including further cardiovascular assessments (including cardiac MRI in a proportion); DEXA scans; OCT; remote measures of cognition, movement and sleep; an expanded suite of fluid biomarker ; and proteomics (Somascan and shortly OLINK).

**Results and Conclusion:**

Work from the Insight 46 study has explored how life course factors including (but not limited to) childhood education, social circumstances, obesity, exercise, head injury, diabetes, infection, hearing, olfaction, vascular risk, menopause, physical capability, emotional and economic adversity, mental health, sleep, and shift working influence later‐life brain health. Key findings will be presented, and opportunities for accessing/applying for study data and collaboration will be discussed.

